# Behandlung der Psoriasis mit Secukinumab: Praxisrelevante Hinweise

**DOI:** 10.1007/s00105-021-04871-9

**Published:** 2021-08-20

**Authors:** Andreas Körber, Matthias Augustin, Frank Behrens, Sascha Gerdes, Ralph von Kiedrowski, Knut Schäkel, Michael Sticherling, Dagmar Wilsmann-Theis, Johannes Wohlrab, Jan-Christoph Simon

**Affiliations:** 1 Dermatologische Privatpraxis, Essen, Deutschland; 2 Institut für Versorgungsforschung in der Dermatologie und bei Pflegeberufen (IVDP), Universitätsklinikum Hamburg-Eppendorf, Hamburg, Deutschland; 3 Rheumatologie, Medizinische Klinik 2, Universitätsklinikum Goethe-Universität, Frankfurt am Main, Deutschland; 4 Psoriasis-Zentrum, Klinik für Dermatologie, Venerologie und Allergologie, Universitätsklinikum Schleswig-Holstein Campus Kiel, Kiel, Deutschland; 5 Dermatologische Spezialpraxis für chronisch-entzündliche System-Dermatosen, Dermato-Onkologie und Allergologie, Selters (Westerwald), Deutschland; 6 Hautklinik, Universitätsklinikum Heidelberg, Heidelberg, Deutschland; 7 Hautklinik und Psoriasiszentrum, Universitätsklinikum Erlangen, Deutsches Zentrum Immuntherapie, Erlangen, Deutschland; 8 Klinik und Poliklinik für Dermatologie und Allergologie, Universitätsklinikum Bonn, Bonn, Deutschland; 9 Universitätsklinik für Dermatologie und Venerologie, Martin-Luther-Universität Halle-Wittenberg, Halle, Deutschland; 10 Klinik und Poliklinik für Dermatologie, Venerologie und Allergologie, Universitätsklinikum Leipzig AöR, Philipp-Rosenthal-Str. 23, 04103 Leipzig, Deutschland

**Keywords:** Infektionen, Komorbidität, Perioperatives Management, Biologika, Besondere Situationen, Infections, Comorbidity, Perioperative management, Biological products, Special situations

## Abstract

**Hintergrund:**

Die mittelschwere bis schwere Psoriasis vulgaris kann wirksam mit immunmodulierenden Biologika wie dem Interleukin-17A-Inhibitor Secukinumab behandelt werden. In der Praxis stellt sich jedoch oft die Frage nach dem Vorgehen in besonderen Situationen, beispielsweise bei Infektionen, Komorbidität, Schwangerschaft oder operativen Eingriffen.

**Ziel der Arbeit:**

In diesem Konsensdokument deutscher Psoriasisexperten sollen in Ergänzung zu den aktuellen Leitlinien häufige Fragen aus dem Therapiealltag zur Behandlung der Psoriasis mit Secukinumab beantwortet werden.

**Methoden:**

In einem virtuellen Expertentreffen im Mai 2020 wurden auf Grundlage von Erfahrungen der Teilnehmer und aktueller Literatur praxisrelevante Aspekte der Behandlung der Psoriasis erörtert. Darauf basierend wurde ein Konsensdokument verfasst.

**Ergebnisse:**

Die vorliegende Arbeit bietet praktische Hinweise zur Anamnese einschließlich der Erfassung von Vortherapien, Schweregrad der Psoriasis und Begleiterkrankungen vor Beginn einer Therapie mit Secukinumab. Ferner wird auf das Vorgehen bei Impfungen, Infektionen, operativen Eingriffen, Sondermanifestationen der Psoriasis und Komorbiditäten einschließlich vorbestehenden Autoimmunerkrankungen und Tumorerkrankungen unter Therapie mit Secukinumab eingegangen. Auch Fragen zur Familienplanung und zu gesundheitspolitischen Regularien werden diskutiert.

**Diskussion:**

Die in diesem Konsensdokument zusammengefassten unterstützenden Empfehlungen zur Behandlung der Psoriasis mit Secukinumab sollen dazu beitragen, für die Patienten eine optimale Therapie zu erreichen und ihre Lebensqualität zu verbessern.

## Abstract (en)

**Background:**

Moderate to severe plaque psoriasis can be treated effectively with immunomodulating biologicals such as the interleukin-17A inhibitor secukinumab. In practice, however, questions often arise as to how to proceed in special situations, such as infections, comorbidity, pregnancy, or surgery.

**Objectives:**

To address frequent questions about the treatment of plaque psoriasis with secukinumab in a consensus document of German psoriasis experts that supplements current guidelines.

**Methods:**

In a virtual expert meeting in May 2020, practical aspects of the treatment of psoriasis were discussed based on the experience of the participants and on current literature. The results of this discussion were summarized in the present consensus document.

**Results:**

This article provides practical guidance on case history, documentation of previous therapies, severity of psoriasis, and comorbidities before starting therapy with secukinumab. For patients treated with secukinumab, the course of action in case of vaccinations, chronic or acute infections, surgical interventions, special manifestations of psoriasis, and comorbidities including history of cancer and autoimmune disorders is discussed. Questions regarding family planning and health policy regulations are also addressed.

**Discussion:**

The recommendations for the treatment of psoriasis with secukinumab summarized in this consensus document may contribute to achieve optimal therapy for patients and to improve their quality of life.

Die mittelschwere bis schwere Psoriasis vulgaris kann wirksam mit immunmodulierenden Biologika wie dem Interleukin-17A-Inhibitor Secukinumab behandelt werden [18, 19]. Die Indikationsstellung berücksichtigt die individuellen gesundheitlichen Voraussetzungen der Patienten wie akute oder chronische Infektionen, Komorbiditäten einschließlich Autoimmun- und Tumorerkrankungen sowie Situationen wie Schwangerschaft oder geplante operative Eingriffe. In einem virtuellen Treffen deutscher Psoriasisexperten aus Klinik und Praxis im Mai 2020 wurden diese und weitere praxisrelevante Aspekte in Bezug auf die Therapie mit Secukinumab erörtert. Die differenzialtherapeutische Wahl des Medikaments bei Vorliegen verschiedener Therapieoptionen wurde hierbei nicht diskutiert. Auf Grundlage der Erfahrung der Experten sowie des Literaturhintergrunds bieten die folgenden Empfehlungen in Ergänzung zu aktuellen Leitlinien eine Hilfe bei Anamnese, Therapie und Management der Psoriasis.

## Anamnese und Laborkontrolle

Voraussetzungen der Indikationsstellung für eine Therapie der Psoriasis vulgaris mit Secukinumab sind:

- Vorliegen einer Psoriasis vulgaris (ICD10 L40.0); auch das Vorliegen einer Psoriasisarthritis kann eine Indikation zur Therapie mit Secukinumab sein, ist aber nicht Gegenstand des vorliegenden Manuskriptes,
- Ermittlung des Schweregrads (mittelschwer bis schwer: ICD10 L40.70!) anhand Psoriasis Area and Severity Index (PASI) und/oder Body Surface Area (BSA) > 10 sowie Dermatology Life Quality Index (DLQI) > 10,
- spezielle klinische Situationen (wie besondere Lokalisation oder starker Nagelbefall) stellen Upgrade-Kriterien bei insgesamt geringem Hautbefall dar [17],
- Ausschluss von Kontraindikationen wie Schwangerschaft, klinisch relevanten aktiven Infektionen (wie aktiver Tuberkulose) oder chronisch entzündlichen Darmerkrankungen (relative Kontraindikation),
- Abklärung chronischer oder rezidivierender Infektionen; bei Vorliegen sollte Secukinumab nur mit Vorsicht angewendet werden,
- Ausschluss einer Überempfindlichkeit gegen den Wirkstoff oder einen weiteren Bestandteil des Medikaments.

Darüber hinaus sollen gemäß Leitlinien Krankheitsbeginn, -dauer und -schweregradverlauf, Familienanamnese, Hinweise auf eine Psoriasisarthritis (PsA), individuelle Triggerfaktoren, Vortherapien, Impfstatus, Infektions- und Tumoranamnese, Komorbidität und Familienplanung erfasst werden [18, 19]. Insbesondere in Bezug auf die letzten 5 Punkte ist ggf. vor Behandlungsbeginn eine besondere Patientenaufklärung erforderlich. Die Anamnese der Vortherapien sollte strukturiert erfolgen und möglichst zu jedem Wirkstoff Dosis, Zeitpunkt von Therapiebeginn und -ende sowie Abbruchgrund erfassen. Vor Beginn der Therapie mit Secukinumab sollten Begleiterkrankungen (z. B. chronisch entzündliche Darmerkrankungen) sowie chronische oder rezidivierende Infektionen in der Vorgeschichte (u. a. Tuberkulose, Hepatitis B) erfragt und aktive Infektionen sowie eine Schwangerschaft ausgeschlossen werden. Spezifische Laboruntersuchungen während der Therapie mit Secukinumab sind gemäß Fachinformation nicht erforderlich [10]. Vor Therapiebeginn, 4 und 12 Wochen danach sowie alle 3 Monate während des weiteren Therapieverlaufs empfiehlt die aktuelle Psoriasisleitlinie dennoch ein Differenzialblutbild und die Bestimmung der Leberwerte.

Zur Ermittlung des Komorbiditätsrisikos beim ersten Patientenkontakt steht eine Checkliste mit Handlungsempfehlungen zur Verfügung (*Supplement*) [32], die sich am bundesweiten Konsens zur Früherkennung von Komorbidität orientieren [20]. Alle 3 bis 6 Monate sollten der PASI und die Lebensqualität mittels DLQI erfasst werden. Insbesondere nach der Induktionsphase mit Secukinumab (ca. 12 Wochen) sollten diese Parameter genutzt werden, um ein ausreichendes Therapieansprechen zu prüfen.

## Wechselwirkungen

Biologika wie Secukinumab werden nicht über das Enzymsystem der Leber metabolisiert und benötigen für ihre Clearance keine Transporter. Von einer direkten Wechselwirkung mit Small-Molecule-Medikamenten einschließlich Methotrexat (MTX) und/oder Kortikosteroiden ist nicht auszugehen. Eine Kombination von Secukinumab mit anderen immunmodulierenden Biologika, die ein erhöhtes Infektionsrisiko zur Folge haben, wird aufgrund unzureichender Studienlage nicht empfohlen. Positive Einzelfallberichte zur Kombination mit Apremilast liegen vor [23]. Falls eine Kombination verschiedener Biologika im Einzelfall notwendig erscheint, sollte dies beispielsweise im Rahmen eines interdisziplinären Entzündungsboards abgestimmt werden.

## Impfungen

Patienten unter immunmodulierender Therapie sollten gemäß Empfehlungen der Ständigen Impfkommission (STIKO) gegen Influenza, Pneumokokken und Meningokokken geimpft sein [25]. Die Autoren empfehlen darüber hinaus eine Impfung gegen Haemophilus influenzae b (Hib). Impfungen mit inaktivierten und Totimpfstoffen sind unter Secukinumab-Therapie ohne Weiteres möglich und sollten im Idealfall in der Mitte eines Behandlungsintervalls erfolgen. Studien haben gezeigt, dass Secukinumab die Impfantwort auf eine Influenza- oder Meningokokkenimpfung nicht negativ beeinflusst [5, 11]. Im Gegensatz dazu ist eine Impfung mit Lebendimpfstoff unter immunmodulierender und potenziell die Infektionsabwehr beeinflussender Biologikatherapie grundsätzlich kontraindiziert (Übersicht in Tab. 1). Lebendimpfungen sollten mindestens 4 Wochen vor Therapiebeginn erfolgt sein oder frühestens 2 Monate nach Therapieunterbrechung bzw. -ende durchgeführt werden [30]. Die Unterscheidung zwischen Lebend- und Tot- bzw. inaktivierten Impfstoffen ist auch für neu entwickelte Impfstoffe beispielsweise gegen COVID-19 zu berücksichtigen. RNA(Ribonukleinsäure)- und Vektor-basierte Impfstoffe können Totimpfstoffen gleichgesetzt werden. Die Therapie mit Secukinumab stellt daher keine Kontraindikation für die Impfung gegen COVID-19 dar.

**Table 1:** 

Impfung gegen	Impfstofftyp	Impfung während Therapie
*Haemophilus influenzae b (Hib)*	*Totimpfstoff*	*Möglich*^a^
*Meningokokken*	*Totimpfstoff*	*Möglich*^a^
*Pneumokokken*	*Totimpfstoff*	*Möglich*^a^
*Diphtherie-Tetanus-Pertussis*	*Totimpfstoff*	*Möglich*
*Hepatitis B*	*Totimpfstoff*	*Möglich*^a^
Herpes Zoster	*Totimpfstoff*	*Möglich*^a^
	**Lebendimpfstoff**	**Kontraindiziert**
*Humane Papillomviren (HPV)*	*Totimpfstoff*	*Möglich*^a^
*Frühsommermeningoenzephalitis (FSME)*	*Totimpfstoff*	*Möglich*^a^
Influenza	*Totimpfstoff*	*Möglich*^a^
	**Lebendimpfstoff**	**Kontraindiziert**
**Masern-Mumps-Röteln (MMR)**	**Lebendimpfstoff**	**Kontraindiziert**
**Varizellen**	**Lebendimpfstoff**	**Kontraindiziert**
Reiseimpfungen		
*Cholera*	*Totimpfstoff*	*Möglich*^a^
**Gelbfieber**	**Lebendimpfstoff**	**Kontraindiziert**
*Hepatitis A*	*Totimpfstoff*	*Möglich*^a^
*Japanische Enzephalitis*	*Totimpfstoff*	*Möglich*^a^
Typhus	*Totimpfstoff*	*Möglich*^a^
	**Lebendimpfstoff**	**Kontraindiziert**

^a^Eine Impfung mindestens 2 und idealerweise 4 Wochen vor Therapiebeginn ist zu bevorzugen

## Infektionen

Vor Beginn einer Therapie mit Secukinumab sollten eine Tuberkulose und mögliche Infektionen mit Hepatitis B oder C sowie bei Vorliegen entsprechender Risikofaktoren auch HIV(„human immunodeficiency virus“)- oder weitere chronische Infektionen abgeklärt werden. Bei klinisch relevanten, aktiven Infektionen besteht eine Kontraindikation. Bei Patienten mit einer chronischen Infektion oder einer rezidivierenden Infektion in der Vorgeschichte sollte Secukinumab mit Vorsicht angewendet werden. Das Vorliegen einer Tuberkulose sollte vor Therapiebeginn mittels Thoraxröntgenaufnahme und IGRA(Interferon Gamma Release Assay)-Test beurteilt werden. Bei Nachweis einer latenten Tuberkulose sollte erwogen werden, 1 Monat vor Therapiebeginn eine Chemoprävention mittels Isoniazid (INH) zu beginnen und diese für insgesamt 9 Monate fortzuführen. Alternativ ist eine Kombinationstherapie mit INH und Rifampicin (RMP) für insgesamt 3 bis 4 Monate bzw. bei INH-Resistenz oder -Unverträglichkeit eine RMP-Monotherapie über 4 Monate möglich [24].

Unter Behandlung mit Secukinumab können vermehrt leichte oder mittelschwere Infektionen der oberen Atemwege sowie Kandidosen auftreten, die jedoch in der Regel keinen Behandlungsabbruch erfordern und auf Standardbehandlung ansprechen [18]. Von einer klinisch relevanten Steigerung des Risikos schwerer bakterieller Infektionen unter Secukinumab wird nicht ausgegangen. Bei Auftreten von Infektionen, insbesondere bei leichten akuten Infektionen, besteht kein grundsätzlich erhöhter Bedarf für eine antibiotische Therapie. Die Entscheidung über Einleitung einer antibiotischen Therapie sollte anhand der individuellen Situation des Patienten unabhängig von der Secukinumab-Therapie getroffen werden.

Bei akuten fieberhaften Infektionen sollte die Therapie pausiert werden. Abhängig von Allgemeinzustand und individueller Situation des Patienten kann in Absprache mit dem behandelnden Arzt frühestens 48 h nach Fieberfreiheit eine Fortsetzung der Therapie erwogen werden. Der Zeitpunkt der ersten Gabe nach Fieberfreiheit kann als Beginn eines neuen Behandlungsintervalls betrachtet werden.

Die Inzidenz viraler Infektionen der Atemwege und grippaler Infekte war in einer aktuellen Datenanalyse unter Secukinumab gegenüber Placebo nicht erhöht [7]. Bezüglich SARS-CoV‑2 empfiehlt die Expertengruppe von BVDD (Berufsverband der Deutschen Dermatologen e. V.) und DDG (Deutsche Dermatologische Gesellschaft e. V.), bei positiv auf das Virus getesteten Patienten ein Herauszögern der Secukinumab-Therapie für die Dauer der mittleren Inkubationszeit von SARS-CoV‑2 zu erwägen. Beim Auftreten von COVID-19-Symptomen sollte die Therapie bis zu deren Abklingen pausiert werden [2].

## Perioperatives Management

Je nach Art und Umfang eines operativen Eingriffs kann eine Biologikatherapie mit einem erhöhten Infektionsrisiko assoziiert sein. Hierzu liegen allerdings nur wenige Studiendaten und keine deutschen Leitlinienempfehlungen vor [18]. Die Entscheidung über eine Unterbrechung der Therapie mit Secukinumab vor operativen Eingriffen sollte neben patientenbezogenen Parametern wie Alter, Begleiterkrankungen und ggf. bereits bestehenden Infektionen auch das mit dem Eingriff verbundene Infektionsrisiko sowie die zeitliche Planbarkeit des Eingriffs berücksichtigen. Als Hochrisikoeingriffe in diesem Kontext sind insbesondere Notfalloperationen sowie Eingriffe an Herz, Thorax und Gefäßen zu sehen, während dermatologische Operationen in der Regel als Eingriffe mit mittlerem oder eher niedrigem Risiko einzustufen sind (Tab. 2; [6]).

**Table 2:** 

Risiko	Art des Eingriffs
**Hoch**	Notfalloperation
	Größere Thoraxoperation
	Herzchirurgischer Eingriff
	Eingriff an der Aorta oder größerer gefäßchirurgischer Eingriff
	Peripherer arterieller Eingriff
	Operationszeit von über 4 h
Moderat	Orthopädischer Eingriff
	Urologischer Eingriff
	Unkomplizierter Eingriff an Thorax oder Abdomen
	Unkomplizierter Eingriff an Kopf oder Hals
	Karotisendarteriektomie
	Eingriff an der Prostata
*Gering*	Endoskopie
	Bronchoskopie
	Hysteroskopie
	Zystoskopie
	Dermatochirurgische Eingriffe
	Brustbiopsie bzw. -exzision
	Ophthalmologischer Eingriff
	Hautbiopsien

Bei elektiven Eingriffen mit mittlerem bis hohem Risiko wird eine Unterbrechung der Secukinumab-Therapie in einem zeitlichen Abstand von etwa 3 Halbwertszeiten (12 Wochen) vor dem Eingriff empfohlen. Bei Eingriffen mit geringem Risiko kann hingegen meist auf eine Therapieunterbrechung verzichtet werden. Notfalloperationen sind jederzeit ohne besondere vorhergehende Maßnahmen in Bezug auf die Secukinumab-Therapie durchführbar, wobei das möglicherweise erhöhte Infektionsrisiko beachtet werden muss.

Eine kleinere retrospektive Studie zum Risiko postoperativer Komplikationen unter Biologikatherapie bei Psoriasis oder PsA kam zu dem Schluss, dass eine Therapieunterbrechung keinen Unterschied in Bezug auf Infektionsrisiko oder Wundheilungsstörungen mit sich bringt, jedoch signifikant mit einer Symptomverschlechterung der Psoriasis bzw. PsA assoziiert ist [4]. Die möglichen Vor- und Nachteile einer Therapieunterbrechung sind daher abhängig von Eingriff und individueller Patientensituation gegeneinander abzuwägen.

## Psoriasisarthritis und Sondermanifestationen

Die Therapie eines Psoriasispatienten mit gleichzeitiger PsA sollte beiden Krankheitsbildern gerecht werden. Bei Patienten mit PsA und gleichzeitiger mittelschwerer bis schwerer Plaquepsoriasis oder solchen, die auf TNF(Tumor-Nekrose-Faktor)-Inhibitoren unzureichend angesprochen haben, beträgt die Secukinumab-Dosis 300 mg [10]. In der klinischen Phase-III-Studie zum Vergleich von Secukinumab mit dem TNF-Blocker Adalimumab bei Patienten mit PsA wurde gezeigt, dass Secukinumab bei Patienten mit begleitender Psoriasis vulgaris (BSA ≥ 3) zu einem besseren PASI-100-Ansprechen führte (*p* = 0,0007), obwohl die Studie in Bezug auf die PsA ihren primären Endpunkt einer signifikant überlegenen Wirksamkeit knapp verfehlte. Secukinumab war zudem mit einer höheren Therapieadhärenz verbunden als Adalimumab [15].

Zu den Sondermanifestationen der Psoriasis vulgaris gehören Nagel‑, Kopfhaut-, genitale und palmoplantare Psoriasis. Die signifikante Wirksamkeit von Secukinumab konnte in jeweils randomisierten, doppelblinden, placebokontrollierten Phase-III-Studien nachgewiesen werden [3, 12, 22]. Langzeitdaten aus der Extensionsphase der Studien zur Nagel- und palmoplantaren Psoriasis belegen die andauernde Wirksamkeit in der Therapie der einzelnen Sondermanifestationen [13, 21].

Die generalisierte Psoriasis pustulosa (GPP) und die Pustulosis palmoplantaris (PPP) stellen eigenständige Krankheitsbilder dar (ICD10 L40.1 bzw. L40.3). Bei der GPP erreichten 83,3 % der Patienten in einer offenen Phase-III-Studie eine Symptomverbesserung innerhalb von 16 Wochen unter Secukinumab-Therapie [14]. Patienten mit PPP profitierten in einer randomisierten kontrollierten Studie von einer Behandlung mit Secukinumab in Form einer Verbesserung der Lebensqualität und des Palmoplantar Pustulosis Area and Severity Index (PPPASI) über 52 Wochen, obwohl der primäre Endpunkt der Studie in Form einer Verbesserung des PPPASI um 75 % innerhalb von 16 Wochen nicht erreicht wurde [16]. Secukinumab ist nur in Japan für die Behandlung von GPP und PPP zugelassen.

## Familienplanung

Eine Anwendung von Secukinumab während der Schwangerschaft ist gegenwärtig nicht zugelassen [10]. Es wird empfohlen, dass Frauen im gebärfähigen Alter während und für mindestens 12 Wochen (entsprechend 3 Halbwertszeiten) nach Ende der Secukinumab-Therapie eine zuverlässige Verhütungsmethode anwenden. Dennoch liegen Echtweltdaten zu ungeplanten Schwangerschaften unter Behandlung mit Secukinumab vor (Exposition im ersten Trimester). Es wurde keine höhere Anzahl von kongenitalen Malformationen oder Spontanaborten beobachtet als in der Gesamtbevölkerung [31]. Von einer diaplazentaren Übertragung von Immunglobulinen (einschließlich Secukinumab) an das ungeborene Kind ist während der ersten 2 Trimester nicht auszugehen. Die Autoren sehen es daher nicht als erforderlich an, eine unter Therapie mit Secukinumab eintretende Schwangerschaft abzubrechen. Eher kann eine engmaschige klinische und sonographisch gynäkologische Verlaufskontrolle der Patientin und des Fetus bis zur Entbindung erfolgen. Die Therapie sollte in diesem Fall jedoch ausgesetzt werden.

Die Anwendung von Secukinumab während der Stillzeit ist nicht zugelassen [10]. Die Wirkung von Secukinumab auf die Fertilität des Menschen wurde nicht untersucht. Tierexperimentelle Studien ergaben jedoch keine Hinweise auf nachteilige Wirkungen [10]. Daher wird nicht von negativen Auswirkungen auf die Fertilität ausgegangen. Bei Männern mit aktuellem Kinderwunsch ist eine Unterbrechung der Therapie nicht erforderlich.

## Gesundheitspolitische Regularien

Zur Einhaltung gesundheitspolitischer Regularien in der Verschreibungspraxis ist es wichtig, Mindeststandards bei der Dokumentation zu wahren (Tab. 3). Eine Dokumentation ist dann ausreichend, wenn auch ein Außenstehender die Therapieentscheidung nachvollziehen kann. Bezogen auf die Therapie mit Secukinumab bedeutet dies vor Therapiebeginn eine entsprechend der ICD10-Kodierung dokumentierte Diagnose sowohl der Psoriasis vulgaris (L40.0) als auch des erforderlichen Schweregrades (L40.70!) oder eines Upgrade-Kriteriums sowie eine Dokumentation der Scores, anhand derer der Schweregrad bestimmt wurde. Neben BSA und/oder PASI schließt dies auch ausdrücklich den DLQI ein [27]. Darüber hinaus sollten Begleiterkrankungen angemessen dokumentiert werden. Bei der Therapiebeschreibung ist auf eine dokumentierte Indikation zur Systemtherapie (Z51.88) zu achten. Zum Nachweis entsprechend Wirtschaftlichkeitsgebot (§ 12 SGB [Sozialgesetzbuch] V), dass die Behandlung wirtschaftlich, ausreichend, notwendig und zweckmäßig (WANZ) ist, sollte ein klar definiertes Therapieziel wie beispielsweise eine Verringerung des PASI entsprechend Leitlinie formuliert werden. Wichtig ist grundsätzlich auch die Dokumentation etwaiger Vortherapien und individueller Faktoren, sofern zutreffend. Die von einigen großen Krankenkassen geforderte Minimaldokumentation ist zudem im „Vertrag zur besonderen Versorgung in der Indikation Psoriasis“ nach § 140a SGB V einzusehen. Dieser entspricht der Dokumentation im Deutschen Psoriasis-Register PsoBest, die als hinreichend für die Dokumentationspflicht in der Systemtherapie angesehen wird [1]. Eine angemessene Dokumentation lässt sich daher mit vertretbarem Aufwand umsetzen.

**Table 3:** 

Diagnose Psoriasis vulgaris (ICD10 L40.0)
Schweregrad mittelschwer bis schwer (ICD10 L40.70!)
Scores (BSA/PASI *und* DLQI)
Gegebenenfalls Upgrade-Kriterien benennen
Begleiterkrankungen (ICD10-kodiert)
Indikation zur Systemtherapie (Z51.88)
Klar definiertes Therapieziel
Gegebenenfalls Vortherapie(n) und Grund für deren Beendigung (primäres oder sekundäres Versagen, Kontraindikationen, Unverträglichkeit)
Gegebenenfalls individuelle Faktoren

*BSA* Body Surface Area, *PASI* Psoriasis Area and Severity Index, *DLQI* Dermatology Life Quality Index

Die Prüfung der Wirtschaftlichkeit einer Verschreibung stellt sich bundesweit sehr heterogen dar. Bundeseinheitliche Vereinbarungen zwischen pharmazeutischen Unternehmen und dem Spitzenverband der Kassen stehen jedoch über regionalen Vereinbarungen. Eine anhand einer guten Dokumentation begründete Praxisbesonderheit bietet deshalb eine hohe Verordnungssicherheit.

## Vorbestehende Autoimmunerkrankungen

Es wird nicht davon ausgegangen, dass eine Therapie mit Secukinumab Autoimmunerkrankungen (u. a. Kollagenosen) auslösen oder verstärken kann. Bei Patienten mit multipler Sklerose (MS), systemischem Lupus erythematodes (SLE) oder Guillain-Barré-Syndrom (GBS) bestehen keine Einschränkungen. Erhöhte Spiegel antinukleärer Antikörper (ANA) vor Therapiebeginn stellen kein erhöhtes Risiko für den Einsatz von Secukinumab dar und sind keine Kontraindikation. Es wurden Fälle von Neuauftreten oder Exazerbationen von chronisch entzündlichen Darmerkrankungen unter Secukinumab berichtet. Bei einer bestehenden chronisch entzündlichen Darmerkrankung wird unabhängig von einer möglichen autoimmunen Ursache dieser Erkrankung eine Therapie mit Secukinumab nicht empfohlen. Wenn ein Patient Zeichen und Symptome einer chronisch entzündlichen Darmerkrankung entwickelt oder eine Exazerbation einer bestehenden chronisch entzündlichen Darmerkrankung auftritt, sollte die Behandlung mit Secukinumab beendet und geeignete medizinische Therapiemaßnahmen sollten eingeleitet werden.

## Wichtige Begleiterkrankungen

**Figure 1:**
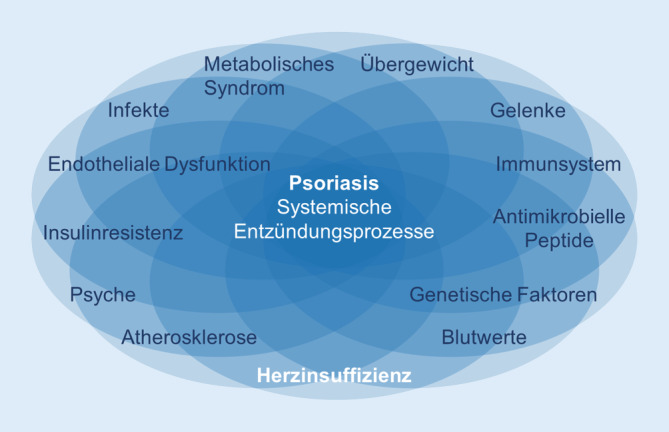

Komorbidität kann bei der Psoriasis sehr unterschiedlich ausgeprägt sein (Abb. 1). Konkrete Handlungsempfehlungen für bestimmte Begleiterkrankungen liegen vor [20]. Besonders häufig und erwähnenswert aufgrund ihrer gegenseitigen Beeinflussung sind das metabolische Syndrom, insbesondere Adipositas, sowie endotheliale Dysfunktion und Atherosklerose. Diese Begleiterkrankungen können durch systemische Entzündungsprozesse verstärkt werden, die im Zusammenhang mit der Psoriasis auftreten und durch Interleukin-17A (IL-17A) gesteuert werden. Eine aktuelle Übersichtsarbeit fasst dies anschaulich zusammen [28]. Eine Anti-IL-17A-Therapie wie Secukinumab könnte sich daher auf diese Begleiterkrankungen positiv auswirken.

So zeigt eine aktuelle Beobachtungsstudie einen Zusammenhang zwischen einer Anti-IL-17A-Therapie und signifikantem Rückgang nicht kalzifizierter Koronarplaques um 12 % nach 1 Jahr. Unter Anti-TNF- bzw. Anti-IL-12/23-Therapie wurde ein Rückgang um 5 bzw. 2 % beobachtet [9]. In einer randomisierten klinischen Studie aus Deutschland wurde gezeigt, dass eine Anti-IL-17A-Therapie mit 300 mg Secukinumab gegenüber Placebo die Endothelfunktion nach 52 Wochen verbessert (*p* = 0,0022), obwohl der primäre Endpunkt einer signifikanten Verbesserung nach 12 Wochen nicht erreicht wurde [29].

Auch auf den Glukosestoffwechsel und damit auf Übergewicht und Adipositas könnte sich eine IL-17A-Blockade durch die Interaktion der Signalwege von IL-17A und Insulinrezeptor positiv auswirken [28]. Bei vorbestehender Nieren- oder Leberinsuffizienz zeigten sich im klinischen Alltag bislang keine Signale für deren Verschlechterung unter Secukinumab-Therapie.

## Tumorerkrankungen

Eine aktuelle systematische Übersichtsarbeit zeigt bei Psoriasispatienten ein insgesamt leicht erhöhtes Risiko für Tumorerkrankungen, jedoch keinen Zusammenhang mit einer Therapie mit immunmodulierenden Biologika wie Secukinumab [26]. Auch in klinischen interventionellen und nichtinterventionellen Studien sowie Registerdaten wurden speziell zu Secukinumab keine erhöhten Raten an erstmanifesten malignen Tumoren im Vergleich zu Placebo oder aktiven Komparatoren beobachtet [8]. Bei einer bestehenden oder aus der Anamnese bekannten malignen Erkrankung ist eine Therapie mit Secukinumab entsprechend Zulassung und deutscher Leitlinie möglich [10, 18], während die europäische Leitlinie maligne und lymphoproliferative Erkrankungen als relative Kontraindikation einstuft [19]. Bei Patienten mit einer onkologischen Erkrankung, die sich noch in der Tumornachsorge befinden, sollte mit den behandelnden Kollegen eine Absprache bezüglich der Therapie mit Secukinumab erfolgen. Bei Patienten mit erhöhtem Risiko für maligne Tumoren bestehen keine prinzipiellen Einwände gegen den Einsatz von Secukinumab. Bei Patienten mit aktiver Tumorerkrankung sollte jedoch in konsequenter Zusammenarbeit mit den behandelnden Ärzten stets eine sorgfältige Nutzen-Risiko-Abwägung erfolgen, wobei für den Einsatz bei Patienten mit nichtmelanozytärem Hautkrebs oder Tumorerkrankungen mit geringem Risiko für Komplikationen im Zusammenhang mit der Psoriasis keine Einwände bestehen.

## Fazit für die Praxis

- Vor Beginn einer Therapie mit Secukinumab sollten Vortherapien, Schweregrad der Psoriasis und Komorbiditäten erfasst und dokumentiert werden.
- Autoimmunerkrankungen sind keine Kontraindikation und bedeuten kein erhöhtes Risiko für den Einsatz von Secukinumab.
- Bei chronisch entzündlichen Darmerkrankungen wird eine Therapie mit Secukinumab nicht empfohlen. Bei kardiovaskulären Vorerkrankungen bestehen keine Einschränkungen.
- Impfungen mit inaktivierten und Totimpfstoffen sind unter Therapie möglich. Lebendimpfungen sind kontraindiziert.
- Bei fieberhaften Infektionen sollte die Therapie unterbrochen und frühestens 48 h nach Fieberfreiheit fortgesetzt werden.
- Bei operativen Eingriffen mit geringem Risiko ist meist keine Therapieunterbrechung nötig.
- Eine Anwendung während der Schwangerschaft ist nicht zugelassen. Der Abbruch einer unter Therapie eingetretenen Schwangerschaft ist nicht erforderlich.
- Es wurde kein Zusammenhang zwischen der Anwendung von Secukinumab und Tumorerkrankungen festgestellt. Bei vorbestehender Tumorerkrankung ist eine Therapie in Absprache mit den behandelnden Ärzten ggf. möglich.
